# Women seeking second opinion for symptomatic uterine leiomyoma: role of comprehensive fibroid center

**DOI:** 10.1186/2050-5736-2-3

**Published:** 2014-04-15

**Authors:** Nelly Tan, Timothy D McClure, Christopher Tarnay, Michael T Johnson, David SK Lu, Steven S Raman

**Affiliations:** 1 Department of Radiology, University of California, 757 Westwood Blvd, Los Angeles, CA 90095, USA; 2 Department of Obstetrics and Gynecology, University of California, 757 Westwood Blvd, Los Angeles, CA 90095, USA

**Keywords:** Fibroids, MRgFUS, Fibroid center, Multidisciplinary

## Abstract

**Objective:**

The objective of the study was to describe our early experience with a comprehensive uterine fibroid center and report our results in women seeking a second opinion for management of symptomatic uterine leiomyoma.

**Methods:**

We performed a HIPAA-complaint, IRB-approved retrospective study of women seeking second opinion for management of uterine fibroids at our multidisciplinary fibroid treatment center in a tertiary care facility from July 2008 to August 2011. After a review of patients’ history, physical examination, and magnetic resonance imaging (MRI) findings, treatment options were discussed which included conservative management, uterine-preserving options, and hysterectomy. We performed Fisher’s exact test for categorical variables between the cohort that did or did not undergo a uterine-preserving treatment. Differences were considered significant at *p* < 0.05.

**Results:**

The mean age of the 205 patient study cohort was 43.8 years (SD 7.5). One hundred sixty-two (79.0%) patients had no prior therapy. Based on MRI, one or more fibroids were detected in 178/205 (86.8%), adenomyosis in 8/205 (3.9%), and a combination of fibroid and nonfibroid condition (i.e., adenomyosis, endometrial polyp) in 18/205 (8.8%). In those who desired to transition their care to our institution (*n* = 109), 85 patients underwent 90 interventions: 39 MRgFUS (magnetic resonance-guided high-intensity focused ultrasound surgery), 14 UAE (uterine artery embolization), 25 myomectomies, 8 hysterectomies, 3 polypectomies, and 1 endometrial ablation. Five patients had two procedures. Intramural and subserosal fibroids were most commonly treated with MRgFUS followed by myomectomy and then UAE; in contrast, pedunculated fibroids were frequently managed with myomectomy.

**Conclusions:**

Multidisciplinary fibroid evaluation may facilitate the increase use of less invasive options over hysterectomy for symptomatic fibroid treatment.

## Introduction

Uterine fibroids, or leiomyomas, are benign growths of the uterine smooth muscle estimated to occur in up to 20% to 35% of all reproductive-age women, and by 50 years of age, prevalence increases to 70% among white and 80% among African-American ancestry [1,2]. Up to 30% of women with uterine leiomyoma are symptomatic [3] with abnormal uterine bleeding and/or bulk symptoms such as pressure, fullness, urinary symptoms, and increased abdominal girth. These symptoms can reduce quality of life in addition to impacting fertility and pregnancy [4,5]. Leiomyoma-related signs and symptoms may be influenced by their location, size, and number [6,7].

The available treatment options for women with symptomatic uterine fibroids include surgical techniques such as hysteroscopic myomectomy, laparoscopic myomectomy, robotic-assisted laparoscopy, minimally invasive options such as uterine artery embolization (UAE), laparoscopic or percutaneous radiofrequency ablation, and noninvasive ablative therapies such as magnetic resonance-guided high-intensity focused ultrasound surgery (MRgFUS). Numerous factors including patient preference, desire for future pregnancy, symptoms, and fibroid number, size, and location are carefully assessed in deciding treatment options.

Despite the proliferation of uterine leiomyoma treatment options in the USA, over 60% of symptomatic uterine leiomyomas are treated surgically with up to 70% undergoing hysterectomy [5]. Over US$2 billion is spent annually for leiomyoma-related care, with the majority of the cost related to inpatient hospital stay after surgery [8,9]. There is limited understanding to explain the current treatment patterns of uterine fibroids [5]. It is likely that a complex set of sociodemographic, economic, referral pattern as well as availability of resources contribute to these trends. In standard of care, women are unlikely to receive a comprehensive set of options regarding treatments for fibroid-related symptoms and are usually offered only a narrow range of treatments based on single physician preference.

In a variety of diseases, a multidisciplinary approach improves patient and disease outcomes with reduction in adverse events compared to traditional standard of care [10-13]. These demonstrated improvements in patient care led us to create a multidisciplinary center for patients with uterine fibroids in 2008. The aim of this study was to describe the clinical outcome of our multidisciplinary fibroid center as related to magnetic resonance imaging (MRI) findings and treatment elected by women seeking a second option for prior diagnosis of symptomatic uterine fibroids.

## Materials and methods

### Study setting

This study was approved by the UCLA Office of Human Research Protection Program and is compliant with HIPAA. A single cohort observational study of all patients consecutively evaluated at our multidisciplinary fibroid center at a major tertiary referral facility from 1 July 2008 to 31 August 2011.

### Multidisciplinary fibroid center

At our multidisciplinary fibroid center, each patient with suspected fibroid-related symptoms underwent consultation by both a radiologist and gynecologist. A variety of other physicians including reproductive endocrinologists, internists, and subspecialists acted as consultants to the multidisciplinary fibroid center as necessary. All physicians were employees salaried by the university academic medical group but billed separately for any given consultation. Women were either referred to the multidisciplinary fibroid center by their gynecologists or primary care physicians or self-referred by online search for uterine fibroid treatment for second or third opinions. Other referrals were from friends, family, radio advertisements, and fibroid treatment features on television talk shows. Women with prior imaging were encouraged to mail their studies to our center for review. In patients with prior documented ultrasound (US) imaging positive for uterine fibroids or if the consultation was for a second opinion, a contrast-enhanced pelvic MRI was performed to better characterize the number, size, and position of the fibroids.

Clinic visits included sequential 30-min consultations with a gynecologist specializing in fibroid-related problems and a radiologist specializing in pelvic imaging and intervention. Each physician independently obtained a comprehensive gynecological history. Fibroid-specific history obtained from the women included the presenting symptoms (bulk vs. bleeding), severity of the symptoms, prior treatments, and the women’s desire for future fertility. A routine physical exam of the heart, lungs, and abdomen and pelvis was typically performed by the radiologist and was supplemented by an independent formal pelvic examination performed by the gynecologist. The contrast-enhanced pelvic MR scan was reviewed concomitantly by both the gynecologist and interventional radiologist to determine the number, size, location of the fibroid, juxtaposition to the endometrial cavity, and relationship to surrounding pelvic organs. The pelvic MR scan was also used to subtype each fibroid (classical, hypercellular, or degenerated) to help improve the triage of these lesions. Classical fibroids were of low signal intensity on T2-weighted imaging and enhanced avidly on dynamic contrast-enhanced T1-weighted imaging and were amenable to treatment by all modalities. Hypercellular fibroids had high T2 signal and enhanced avidly on dynamic contrast-enhanced T1-weighted imaging and responded poorly to MRgFUS. Lesions that lack enhancement are thought to have undergone either red or cystic degeneration and would not benefit from either uterine embolization or MRgFUS.

**Figure 1:**
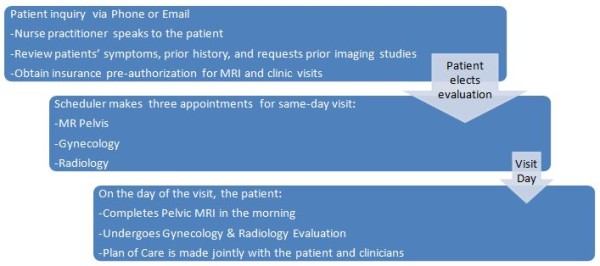
**The flowchart of the process patients take.** It is from the time point they learn about the center to the time when they are seen by the center’s providers.

Information from the history and physical examination, MRI, and patients’ treatment request were used to offer one or more treatment options to each woman. The ultimate treatment decision was made in partnership with the patient with respect to her preference. If a nonfibroid condition was diagnosed, further work-up was then performed on a case-by-case basis by the gynecologists (see Figure 1).

### Magnetic resonance imaging

MRI of the pelvis was performed in prone position with an external pelvic phased array coil for reception using either 1.5-T (Sonata, Avanto, Siemens Medical Solutions, Erlangen, Germany or Horizon LX; GE Medical Systems, Milwaukee, WI, USA) or 3-T scanners (Trio, Vario, Siemens Medical Solutions, Erlangen, Germany). Women were scanned in the prone position to most closely mimic treatment position during MRgFUS. The MRI scans of the pelvis include the following sequences: axial T1 2D spoiled gradient-recalled echo dual-echo (FMPSGPR, GE Healthcare, Milwaukee, WI, USA or FLASH 2D, Siemens Medical Systems, Erlangen, Germany), axial 3D T1 with fat saturation (liver acquisition with volume acceleration (LAVA), gradient echo (GE), or volumetric interpolated breath-hold examination (VIBE); Siemens), axial 2D single-shot T2 (SSFSE, GE, or HASTE; Siemens), axial, coronal, oblique axial, and coronal of uterus multishot T2 (FLARE, GE, or RESTORE; Siemens). After intravenous power injection of 0.1 mmol/kg of gadolinium DTPA (Magnevist, Bayer Healthcare, Berlin, Germany) at 2 cm^3^/s, dynamic sagittal 3D VIBE with fat saturation imaging was performed at 30, 60, 90, and 120 s. Coronal and axial 3D VIBEs with fat saturation were also obtained at 180 and 300 s, respectively. One of two fellowship-trained abdominal imaging radiologists with 14 and 19 years of experience in pelvic MR interpretation reviewed all the studies on a commercially available workstation with high-resolution monitors (Centricity 2.0, GE Healthcare). For each fibroid, the radiologists determine the size, number and location, T2 signal, presence of adenomyosis or endometrial polyps, relative enhancement after intravenous gadolinium, and the dominant arterial supply to the uterus. The adnexa and cervix were evaluated to exclude incidental masses.

### Treatment algorithm

In the multidisciplinary fibroid center, treatment was stratified into medical therapy, MRgFUS, UAE, hysteroscopic resection, myomectomy (laparoscopic, robotic, or open), and hysterectomy. Medical therapy, such as oral contraceptive pills, were used as first-line treatment for women with abnormal uterine bleeding, but women who were refractory to conservative treatment can elect to undergo any of the appropriate therapies. Gonadotropin-releasing hormone (GnRH) was also used to manage abnormal uterine bleeding or to reduce the fibroid size prior to myomectomy or MRgFUS.

MRgFUS is a noninvasive treatment option most indicated in women with a single dominant, classical, nonpedunculated, nondegenerated fibroid less than 10 cm in size and is offered to women who desired future fertility. On a case-by-case scenario, women with more than one fibroid may be offered MRgFUS. Women with hypercellular or degenerated fibroids were considered poor candidates for MRgFUS.

UAE was discussed as a treatment option primarily in women with intermediate-sized (8–10 cm) to large-sized (>10 cm), nonpedunculated, classical or hypercellular, nondegenerated fibroids (>10 cm) or with multiple fibroids (>5). UAE was typically not offered to women who desired future fertility.

With respect to women who chose surgery but desired future fertility or in women with large pedunculated fibroid, myomectomy (robotic-assisted laparoscopic approach, hysteroscopic, or open) was the recommended therapy. Desire for future fertility precluded UAE and hysterectomy, but not myomectomy or MRgFUS.

Hysteroscopic myomectomy was recommended for women with small (<4 cm) intracavitary leiomyomas, especially if they are pedunculated (type 0) or >50% of the circumference is bulging into the endometrium (type 1).

Open or robotic-assisted laparoscopic or laparoscopic hysterectomy was recommended when patients had symptom recurrence after the first-line treatment failed. See Table 1 for the summary of inclusion and exclusion criteria for various treatment therapies.

**Table 1:** Summary of inclusion and exclusion criteria for various treatment therapies

**Treatment**	**Inclusion criteria**	**Exclusion criteria**
MRgFUS	• Single, dominant if <10 cm	• Degenerated or pedunculated
	• Multiple (≤5 fibroids) if <5 cm	• Hyperintense signal on T2-weighted (hypercellular) MRI
	• Desires fertility	
UAE	• Intermediate (5–10 cm) to large (>10 cm) fibroids	• Degenerated or pedunculated
	• Multiple >5 fibroids	• Desires fertility (relative contraindication)
Myomectomy (robotic-assisted, hysteroscopic, or open)	• Desires fertility	• Poor surgical candidate (significant comorbidities)
	• Small (<4 cm) intracavitary	
	• Pedunculated	
Hysterectomy	• Failed first-line treatment	• Poor surgical candidate (significant comorbidities)
		• Desires fertility

### Data

Patient demographics, clinical history, MRI findings, treatment eligibility, and follow-up management outcomes were retrieved from the electronic medical records and recorded in a database by a single medical provider.

### Statistical analysis

Mean and standard deviation were determined for age and number of fibroids; median and range were measured for fibroid size. We performed Fisher’s exact test for categorical variables between the cohort that did or did not undergo uterine-preserving treatments. All analyses were done using the statistical software STATA/SE 11.2 (College Station, TX, USA), and statistics were considered significant at *p* < 0.05.

## Results and discussion

### Results

#### Patients

The study cohort consisted of 205 patients with a mean age of 43.8 years (standard deviation (SD) 7.5). Nearly all patients had received a diagnosis of uterine fibroids from outside clinics based on physical exam and/or ultrasound, and most had been offered hysterectomy from outside facilities for treatment. The most common presenting complaints were bleeding symptoms (41.9%), bulk symptoms (25.6%) or combination bulk and bleeding symptoms (12.7%). The remainder of patients presented with a variety of other symptoms unrelated to either bulk or bleeding (e.g., dyspareunia, abdominal pain, back pain, urinary incontinence, and infertility; 20.0%). One hundred sixty-two (79.0%) patients had not had prior therapy. In 43/205 (21.0%) patients with a history of prior treatment, 27/43 (62.8%) had oral contraceptive pill or gonadotropin-releasing hormone agonist, 9/43 (20.9%) had myomectomy, 2/43 (4.6%) had endometrial ablation, and 5/43 (11.6%) had prior UAE.

#### Treatment recommendations

**Figure 2:**
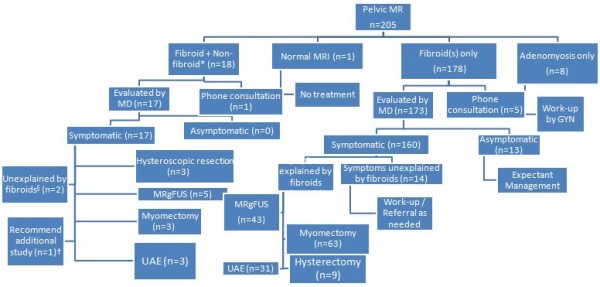
**General treatment algorithm and distribution of recommendations.** General treatment algorithm for women presenting to the comprehensive fibroid center and the distribution of recommendations made based on patients’ symptoms and MRI findings. *Asterisk* denotes nonfibroid conditions existing concurrently with fibroids: adenomyosis (*n* = 13), ovarian cyst endometrioma (*n* = 2), endometrial polyp (*n* = 3). *Section sign* denotes that one patient had urinary retention; the other patient had imaging findings that did not correspond to the patient’s pelvic pain. *Dagger* denotes that hysterosonogram was recommended to evaluate for a possible endometrial polyp. Recommendations to be evaluated at the fibroid center were made when feasible for the patient.

Treatment recommendations were made jointly by the radiologist and gynecologist based on the patients’ symptoms, physical exam findings, MRI findings, and preferences. Although all patients sought therapy for presumed uterine fibroids based on a previous diagnosis, up to 13.2% had either a combination of fibroid and nonfibroid conditions (i.e., adenomyosis, endometrial polyp) in 18/205 (8.8%) or adenomyosis in 8/205 (3.9%) patients (Figure 2). Based on MRI, fibroids were detected on MRI in 178/205 (86.8%) patients. The majority of patients were eligible for treatment with medical or uterine-preserving therapies to manage their symptoms (Figure 2). Hysterectomy was recommended for 9/205 (4.4%) patients (Table 2).

**Table 2:** **Reasons for the** r**ecommended hysterectomy in nine patients**

**Patient**	**Clinical and imaging findings**	**Follow-up care**
1	17-cm fibroid	Open supracervical hysterectomy. Patient had endometriosis seen intraoperatively which was cauterized
2	>7 fibroids, 3–5 cm which increase in size causing dyspareunia	Open supracervical hysterectomy
3	21-cm posterior exophytic fibroid	Open supracervical hysterectomy
4	Multiple fibroids; largest is 7 cm with concomitant adenomyosis	Robotic-assisted laparoscopic supracervical hysterectomy
5	Multiple fibroids; largest is 10 cm	Robotic-assisted laparoscopic supracervical hysterectomy
6	Multiple fibroids; largest is 4 cm with concomitant adenomyosis	No follow-up
7	Patient s/p UAE with peripherally calcified 6-cm fibroid	No follow-up
8	7-cm fibroid extending to the cervix	No follow-up
9	Multiple 6–7-cm fibroids	No follow-up

#### Treatment provided

**Figure 3:**
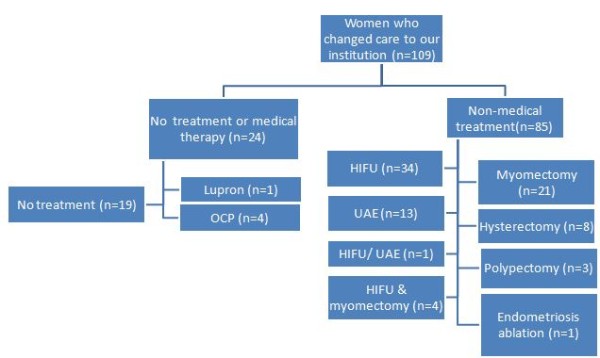
**Treatment provided to women who changed to our institution.** In women who changed their care to our institution after initial consultation, the following describes the treatment provided to the patients based on their preferences, symptoms, and MRI findings.

Of the 205 patients in the study cohort, 109 (53.2%) elected to transfer their care to our institution. Medical therapy or no further treatment was recommended for 24/109 patients (22.0%; Figure 3). A total of 85/109 patients (77.9%) underwent 90 procedures, and 8/109 patients (7.3%) underwent hysterectomy (Figure 3). The distribution of the treatment type provided to the patients who had follow-up care with our institution was based on characterization and localization of uterine pathology by MRI (*p* ≤ 0.01; Table 3). The median size and number of fibroids among 85 patients who underwent 90 procedures were reported in Table 4. Of the myomectomy cohort, 15/25 (60%) patients were managed with minimally invasive surgical procedures (Table 4).

**Table 3:** Distribution of interventions provided based on characterization and localization of uterine pathology by MRI in 88 women

**Diagnosis**		**MRgFUS (*n*** **= 39)**	**UAE (*n*** **= 14)**	**Myomectomy**^**c **^**(*n*** **= 25)**	**Hysterectomy (*n*** **= 8)**	**Endometrial ablation (*n*** **= 1)**	***p *** **value**
Fibroids (subtypes)	Intramural/subserosal (*n* = 60)	31	9	17	3	0	<0.01^d^
	Submucosal (*n* = 17)	7	3	3	3	1	
	Intracavitary (*n* = 12)	4	3	5	0	0	
	Exophytic (*n* = 7)	3	1	2	1	0	
	Pedunculated^a^ (*n* = 11)	0	2	7	0	0	
	Cervical (*n* = 1)	0	0	1	0	0	
Others	Adenomyosis (*n* = 9)	4	2	1	2	0	-
	Endometrial polyp (*n* = 3)^b^	0	0	0	0	0	-

These women underwent nonmedical treatment, while women electing expectant management or medical therapy were not included in this table.

^a^Though UAE is not the standard treatment for pedunculated fibroids, each of the two patients who underwent uncomplicated UAE had multiple (>10) fibroids. Both patients had a nondominant small (<2 cm) pedunculated fibroid seen on MR.

^b^All three patients with endometrial polyps underwent polypectomy.

^c^Open/laparoscopic-assisted robotic myomectomy.

^d^Fisher’s exact test was used.

**Table 4:** Size and number of fibroids in 85 patients who underwent 90 procedures

**Procedures (total)**	**Age**	**Fibroid size (cm)**	**Number of fibroids**	**Miscellaneous**
	**Mean (SD)**	**Median (range)**	**Mean (SD)**	
MRgFUS (*n* = 39)	40.4 (9.5)	7.3 (5–10)	2.6 (2.9)	Two patients had MRgFUS adenomyosis
UAE (*n* = 14)	47.1 (4.7)	7.8 (6.2–8.5)	2.2 (1.1)	Two patients had UAE for isolated adenomyoma
				Two patients were with hypercellular intramural >8 cm fibroids
				Four patients were with >5 fibroids
Myomectomy (*n* = 25)	43.2(6.6)	8.0 (5.4–11)	2.9 (2.2)	Approach: 11 hysteroscopic, 10 open, 2 robotic, 2 laparoscopic myomectomies
Hysterectomy (*n* = 8)	46.0 (5.7)	9.5 (7.8–14.0)	4 (3.7)	Two patients were with isolated adenomyoma
				Approach: two laparoscopic hysterectomies
Polypectomy (*n* = 3)		-	-	-
Endometrial ablation (*n* = 1)		2.2 cm	1	-

Five patients had two procedures.

Five patients had two treatments (4.6%; Table 4). Patient 1, with pressure symptoms and abnormal uterine bleeding who on MRI had an intracavitary lesion and multiple intramural fibroids, had planned a combination of MRgFUS for the intramural lesions and hysteroscopic myomectomy for the intracavitary fibroids. Patient 2 had pressure symptoms and had a 10-cm pedunculated fibroid and a 5-cm intramural fibroid on MRI and underwent a combination of MRgFUS for the intramural fibroid and robotic-assisted laparoscopic myomectomy for the pedunculated fibroid. Patient 3 who presented with pressure and bleeding symptoms had a 10-cm intramural fibroid abutting the endometrial stripe on MRI and underwent MRgFUS with bleeding symptom improvement but had persistent bulk symptoms and subsequently underwent open myomectomy. Patient 4 presented with urinary frequency and had a 10-cm submucosal fibroid on MRI; she elected and underwent MRgFUS with transient symptomatic improvement. Subsequently, the patient underwent open myomectomy due to symptom recurrence. Patient 5 had bulk symptoms and had multiple intramural fibroids. Though not ideal for MRgFUS, this patient was directive about receiving this modality and after explicit risk/benefit counseling underwent MRgFUS. She did not have sufficient improvement in her symptoms and subsequently had UAE with symptom relief.

## Discussion

In this study, we have shown that a multidisciplinary fibroid center model is feasible and provides patients with a full range of treatment options including alternative therapy. On contrast-enhanced MRI, 13.2% of the patients with prior outside diagnosis of uterine leiomyoma had other pathologies on MRI (i.e., adenomyosis and endometrial polyp). The distribution of the procedure type depended on the fibroid size, MR-based subtype (classical, hypercellular or degenerated), location, and concomitant existing uterine pathologies. In our cohort, most patients qualified for uterine-preserving treatment; we suggested hysterectomy in 4.4% of our patients although the majority had been offered hysterectomy at other facilities. Of patients who changed care to our institution, 77.9% were treated with either an image-guided procedure or surgery. MR-guided focused ultrasound was the most common procedure performed followed by myomectomy.

Pron [14] questioned why adoption of newer approaches for treatment of uterine fibroids was lagging despite evidence supporting the safety and efficacy of minimally invasive therapies. She argued that the issue of low adoption was due to the behavioral and practice patterns of clinicians who may be uncomfortable or unfamiliar with nonsurgical options performed by other specialties. Even among gynecologists, adoption of newer, minimally invasive, uterine sparing techniques may be variable. In a study reviewing clinical experience from the UK [15], a survey of gynecologists reported that 11% performed laparoscopic myomectomy and 43% performed hysteroscopic myomectomy. The author argued that the low rate of adoption of the laparoscopic approach was likely due to lack of training, the nonavailability of instruments, as well as worries about prolonged operative time compared to a hysteroscopic approach. Further, although 51% of gynecologists had access to UAE, the median number of patients referred to UAE was only five per year [15]. The results affirmed the underutilization of minimally invasive surgery and nonsurgical options for treatment of uterine fibroids. The underutilization of minimally invasive options was also reported in a study in the USA, where of the 42% of women with fibroids who underwent an intervention, 78% of these women underwent hysterectomy with only 10% undergoing myomectomy, 3% undergoing endometrial ablation, and 4% undergoing multiple therapies [5].

We have shown that gynecologists and interventional radiologists can successfully partner together to provide women with fibroid-related symptoms the widest variety of treatment options possible. In our multidisciplinary fibroid center, women seeking second opinion tended to choose less invasive options for symptomatic fibroid treatment favoring nonsurgical procedures followed by myomectomy (Table 4). Among the myomectomy cohort, 60% were also managed using a laparoscopic, robotic, or hysteroscopic approach over open myomectomy. The results suggest that in the setting of a multidisciplinary center in women seeking second opinion, a less invasive surgical option is preferred even for large or multiple fibroids.

The discrepancy between distribution of treatments among this study’s cohort and those reflecting the national trends may be due to how patients and clinicians learned about emerging fibroid therapies. Ankem [16] surveyed radiologists in Michigan to understand the sources of information radiologists used to learn about UAE. The results demonstrated that while national and regional conferences were the initial source of information for the early adopters, colleagues’ practice patterns were the most important source of information for late adopters. The study argued that work relations and communication among clinicians was the most conducive means for flow of information about UAE. An institution-based practice as well as availability of appropriate resources may explain why newer modalities were widely accepted and used in our clinical practice.

Patients usually uncover information about nontraditional and alternative fibroid therapies from internet sources, and this may also explain why our cohort of patients elected the therapies they did. One study [17] investigated the information-seeking behavior of patients with symptomatic fibroids, specifically in those who learned about and underwent UAE. In this study, 40% learned about UAE from their gynecologist and 60% became aware of UAE through mass media, friends, or family members. Our experience confirmed this result since the vast majority of women were offered only hysterectomy in the community. Additionally, women in this study were asked to rate the helpfulness of an information source in providing information that they needed. The study found that the radiologists were viewed as the most helpful in providing information, followed by the internet, then by gynecologists and other primary care doctors.

MRI is regarded as the definitive modality for uterine fibroid imaging. Although initially expensive, it is the most accurate and reproducible imaging method for detecting, localizing, and characterizing leiomyoma [18,19] Unlike US or CT, MRI can also characterize fibroid subtypes, assess for fibroid degeneration, delineate the presence of gonadal arterial supply to individual fibroids (for UAE planning), and uncover a range of coexisting pathologies such as adenomyosis, polyps, urethral diverticula, and adnexal masses. [20] Thus, MRI enables a comprehensive and objective noninvasive gynecological evaluation to determine whether a patient undergoes laparoscopic myomectomy, UAE, or MRgFUS. It provides an immediate method of triage for a range of problems that often coexist like adenomyosis and endometrial polyps, where in tandem were detected in up to 13% of our cohort with outside US diagnosis of fibroids. MRI has been proven superior to US in diagnosing adenomyosis [21-23]. Although adenomyosis may be treated with UFE, its role was still evolving, and preoperative diagnosis was important for triage. Unlike with US, MRI also enabled a confident diagnosis of degenerating fibroids, which would not benefit from UAE or MRgFUS. Similarly, MRI enabled a confident diagnosis of hypercellular fibroids which respond very poorly to MRgFUS [24]. Thus, the European Society of Human Reproduction and Embryology [25] endorsed MRI as a second-line technique examination for examining morphologic and vascular features of leiomyoma and for triaging treatment options as compared to US primarily due to the higher expense of MRI.

We recognize that the use of MRI as the primary diagnostic tool at our center is not the standard of practice. However, we feel that our practice is a specialized center and that the patient population is unique compared to a typical gynecology or primary care practice. Most women came with known diagnosis of fibroids and came to us seeking specific treatments not available to all practices (i.e., MRgFUS). MRI allowed us to provide the most comprehensive and accurate noninvasive diagnosis to guide appropriate treatment recommendations during multidisciplinary consultation. There has not been a trial of cost-effectiveness of using MRI to triage women with leiomyoma over US as the primary imaging modality. However, Schwartz et al. [26] prospectively evaluated the impact of MRI on treatment decisions for 69 consecutive women scheduled to undergo gynecologic surgery and effect on change in medical expense. The diagnosis was changed in 53% of patients. Of this cohort, 81% had a change in treatment; the most common change was cancellation of surgery. Use of MRI resulted in overall savings per patient. Moreover, Lin et al. demonstrated that people are willing to undergo diagnostic radiology exams—and are willing to pay more—to reduce unnecessary, invasive medical procedures and overall health costs [27].

There were several limitations to our study. One significant potential source of bias was the self-referred nature of our patient population. These women were searching online for uterine-preserving options and would likely choose uterine-preserving treatments, and thus, the results should not be generalized to all patients. Patients also likely came with a preconceived notion for specific treatment, creating another source of selection bias for the study cohort, and thus, the results may not be applicable to the general population. Another bias was in the socioeconomic status of our patients. In general, our patient population had health insurance, and as a result, outcomes and conclusions may not be representative of the general population across all socioeconomic groups. Moreover, our medical center provided all comprehensive treatments, and as such, our findings may not be applicable to community-based practices, which may have limited access to certain treatment such as MRgFUS, robotics, or UAE. We used MRI as the diagnostic study of choice which was not the standard practice. Therefore, we do not advocate MRI for initial presentation and evaluation. However, in women seeking a second opinion for prior diagnosis of uterine leiomyoma at a comprehensive fibroid center, MRI allowed us to provide efficient and definitive diagnosis to direct the patient to appropriate treatments.

Despite the limitations, our study supported the feasibility of establishing multidisciplinary fibroid centers to address women with symptomatic fibroids. With increasing access to the internet and utilization of social media, patients will likely be the driving force to the adoption of less invasive, less radical treatment alternatives across all surgical fields including gynecology as they learn about new and evolving treatment options. In our patient population, most women were candidates for uterine-preserving options and chose to undergo a minimally invasive uterine-preserving treatment.

## Conclusions

Uterine fibroids had been managed by a single subspecialist effectively, typically gynecologists. However, with the advent of multiple therapeutic options for treatment of symptomatic uterine fibroids which are performed by different subspecialists, there is an increasing need for crosstalk between multiple specialties to provide comprehensive options for women. Patients can then decide for themselves which treatment is best suited for their needs and wishes. In the USA, most women are managed with surgical therapy, most commonly hysterectomy. The reasons for this are likely multifactorial including social, economic, geographic, and access to care. To overcome some of the barriers for patients, we started a comprehensive fibroid center in collaboration with gynecology and radiology, and we reported our early experience with the center. Our results demonstrate that patients undergo a wide range of therapy. However, there is a significant preference towards uterine-preserving options. Of these, MR-guided focused ultrasound surgery was the most commonly utilized procedure. Our findings suggest that women desire minimally invasive therapies, and joint effort between gynecology and radiology maybe one option for institutions to improve access to most, if not all, of the therapeutic options available for symptomatic uterine fibroids.

## Abbreviations

MRgFUS: MR-guided focused ultrasound surgery; MRI: magnetic resonance imaging; UAE: uterine artery embolization.

## Competing interests

Nelly Tan was the Focused Ultrasound Surgery Foundation Clinical Fellow in 2012. Timothy D. McClure was the Focused Ultrasound Surgery Foundation Clinical Fellow in 2010. Christopher Tarnay, Michael T. Johnson, David SK Lu, and Steven S. Raman have no competing interests to declare.

## Authors’ contributions

NT participated in the design, data acquisition, analysis, interpretation, and drafting of the manuscript. TDM participated in the design, interpretation, and drafting of the manuscript. CT participated in the design and drafting of the manuscript. MTJ participated in the conception and drafting of the manuscript. DSKL participated in the conception and design and drafting of the manuscript. SSR participated in the conception and design and drafting of the manuscript. All authors read and approved the final manuscript.
